# Impact Strength and Water Uptake Behaviors of Fully Bio-Based PA11-SGW Composites

**DOI:** 10.3390/polym10070717

**Published:** 2018-06-29

**Authors:** Helena Oliver-Ortega, José Alberto Méndez, Francesc Xavier Espinach, Quim Tarrés, Mònica Ardanuy, Pere Mutjé

**Affiliations:** 1 Group LEPAMAP, Department of Chemical Engineering, University of Girona, C/M. Aurèlia Capmany, 61, 17003 Girona, Spain; jalberto.mendez@udg.edu (J.A.M.); joaquimagusti.tarres@udg.edu (Q.T.); pere.mutje@udg.edu (P.M.); 2 Design, Development and Product Innovation, Department of Organization, Business Management and Product Design, University of Girona, C/M. Aurèlia Capmany, 61, 17003 Girona, Spain; francisco.espinach@udg.edu; 3 Department of Materials Science and Metallurgy, Textile Engineering, Polytechnic University of Catalonia, C/Colom, 11, 08222 Terrassa, Spain; monica.ardanuy@upc.edu

**Keywords:** bio-based composites, polyamide 11, lignocellulosic fibers, impact properties, water uptake

## Abstract

Composite materials have attracted the attention of some industrial fields due to their lightness and relatively good mechanical properties. One of these properties is impact strength, essential to ensure the processability and application of these materials under impact conditions. In addition, it is known that water absorption has a plasticizing effect in polymers and polymer composites which can change the properties of such materials and limit their use. Moreover, this effect worsens when hydrophilic reinforcement is used. In this work, the impact and water uptake behavior of totally bio-based composites from polyamide 11 (PA11) and lignocellulosic pine fibers mechanically processed as stone groundwood (SGW) were studied. The impact resistance of PA11 and its composites was higher than expected, obtaining better results than those of polyolefin-based materials. The evaluated mechanical properties and the micrographs showed an adequate interface. The water uptake test showed that PA11 and its composites had non-Fickian and Fickian case I behaviours, respectively. It was found that the maximum water absorbance was similar to that of SGW reinforced polypropylene.

## 1. Introduction

Materials like polymers and polymer composites have attracted the interest of industries like automotive and construction because they are able to show competitive mechanical properties while having comparatively low densities. An example of such materials are fiber reinforced polymers. These composites show noticeable strengths and stiffness, allowing use under higher loads than the neat polymer, while at the same time, showing lower deformations under normal use conditions. The most commonly used properties to evaluate a possible application of these materials are strength and stiffness [1]; hence, lots of papers evaluate these properties under flexural or tensile conditions.

Nonetheless, in a great number of cases, the composites can be subjected to collision during their lifespan; the response of the materials to such collisions has security concerns. Thus, knowing the impact behavior of these materials is crucial for the industry. The impact behavior of a composite material is mainly influenced by the reinforcement content and the quality of the reinforcement-matrix interface [2]. Usually, the impact strength decreases with the percentage of reinforcement [3]. This decrease can be balanced with a good fiber-matrix interface that allows a good transmission and dissipation of the energy [4,5].

The automotive and construction industries are interested in materials with better relative properties, but also in greener or more sustainable ones [6]. In this sense, bio-based polymers and reinforcements are very interesting options. Polyamide 11 (PA11), obtained from castor oil, with comparatively high mechanical performance, good chemical resistance, and durability, is a promising matrix to formulate bio-composites, and an alternative to oil-based polymers [7,8,9]. Moreover, previous studies reported that natural fiber reinforced PA11 composites showed competitive mechanical and thermo-mechanical performance with respect to commodity uncoupled polypropylene-based composites [10,11]. Unlike polyolefin, PA11 can establish hydrogen bonds with the cellulosic fibers, producing good interfaces [12]. These interfaces can be strong enough to allow a good energy transfer under impact loading, but, to the best knowledge of the authors, the literature about this subject is scarce and general in nature [10].

On the other hand, the applications for automotive or construction purposes also involves use under humid environments. The reduction of the mechanical properties of polymeric materials due to moisture adsorption is well established in the literature [13]. This is because the small size and mobility of water molecules facilitates their diffusion to the amorphous phase of the polymers. Moreover, the polar groups in the polymer chains interact with water [14,15]. In the case of natural-fiber reinforced composites, the hydrophilic behavior of natural fibers accelerates the process due to its high capacity to absorb water [16].

Polyamides are hygroscopic polymers, due to the presence of amide groups [17]. Nonetheless, among polyamides, PA11 has a low hydrophilic behavior due to its lower content of amide groups [18]. In addition, due to its low melting temperature, PA11 can be reinforced with lignocellulosic reinforcements, avoiding or limiting fiber degradation [19]. The relatively high glass transition temperature (*T_g_*) of PA11, around 50 °C, can also contribute to inhibiting its water absorption at room temperature due to the low mobility of PA11 chains at temperatures under the *T_g_*. Moreover, the stiffness enhancement of the composite materials provided by the reinforcing fibers can further decrease mobility, also reducing the diffusion of the water molecules into the material.

In this work, the impact properties and the water absorption behavior of PA11 reinforced with different contents of stone groundwood fibers (SGW) were investigated. The impact properties were evaluated using Charpy impact energy specimens. Micromechanical models were used to establish the energy devoted to creating the fracture and its propagation during the impact test. The water absorption behavior was analyzed, determining water uptake of the composite materials under water immersion. Two different water immersion temperatures were studied, and the kinetic parameters of the absorption phenomena were determined.

## 2. Materials and Methods

### 2.1. Materials

Polyamide 11 (Rilsan^®^ BMNO TL) used as bio-based matrix was kindly provided by Arkema S.A. (Colombes, France). Its density is 1.03 g/cm^3^, and its melt volumetric index is 11 cc/10 min at 235 °C and under 2.16 kg.

The mechanical pulp used as reinforcement (SGW), supplied by Zubialde S.A. (Aizarnazabal, Spain), was obtained from pine fibers through a stone groundwood process. Their mean length and diameter are in the micro scale inside the composite material [7,12]. The use of a commercial lignocellulosic reinforcement ensures reliable mechanical, chemical, and morphological properties with low dispersions.

### 2.2. Composite Compounding

Composites with reinforcement contents ranging from 20% to 60% *w*/*w* were produced in a Gelimat Kinetic Mixer (model G5S, Draiswerke, Mahaw, NJ, USA). The compounding and injection processes have been described in previous works [7,12]. The polymer and the fibers were added at low speed and then the speed was increased up to 2500 rpm. The blend was discharged when the mix reached 200 °C. Specimens for the Charpy impact test were obtained by injection-molding following the ASTM D3641 standard. For the water uptake test, tensile samples Type I described in the ASTM D638 standard were also obtained by injection-molding. The injection process was performed in a Meteor-40 injection-molding machine (Mateu and Solé, Barcelona, Spain; clamping pressure: 40 tons). The processing temperature profile was 170–185–200 °C and the maximum pressures used were 75 bars for the volumetric phase and 30 bars for the maintenance pressure.

All the samples were conditioned in a climatic chamber for 48 h at 23 °C and 50%RH according to ISO D618 prior to the test.

### 2.3. Impact Characterization

Charpy impact tests were performed using notched and un-notched specimens using a Resil 5,5 hammer by Ceast instrument (Pianezza, Italy) following ISO 179 standard (Figures S1 and S2 of the Supplementary Materials). The absorbed energy of the material during crack formation and fracture propagation was also determined for the un-notched samples. At least five specimens of each composite were tested.

The calculation of the standard error and the significant figures were based on the rules proposed by Taylor J.R. [20].

### 2.4. Scanning Electron Microscopy (SEM)

Micrographs of the fractured surface of the impact test samples were obtained by scanning electron microscopy (SEM). The images were taken with a Zeiss DSM 960A (Carl Zeiss Iberia, Madrid, Spain); sample preparation required coating with gold.

### 2.5. Hydrophilic Behaviour: Water Contact Angle

The hydrophilic behavior of the studied PA11 and PA11 reinforced with 20%, 50% and 60% *w*/*w* of SGW were determined by means of water contact angle. A DSSA25 drop-shape analyzer from Krüss GmbH (Hamburg, Germany) was used to observe the angle, and was controlled with the Krüss Advance Software. A total of 120 measurements were made during one minute for each sample; the assay was performed at room temperature. The average value was obtained from the test of 3 samples for each specimen.

### 2.6. Immersion Water Uptake Test

The composite specimens were dried at 105 °C for 2 h before their immersion in order to remove any residual moisture. Afterwards, the samples were immersed in distilled water. Two sets were prepared: one at 23 °C, the other at 40 °C. The specimens remained under immersion until saturation. The water uptake was calculated by weight difference of the samples, and the saturation point was determined by constant weight of the samples.

## 3. Results

### 3.1. Impact Strength

Impact strength was obtained by means of a standard Charpy test. In this test, a pendulum hammer is thrown from a measured height to a sample bar. The difference in height or in potential energy before and after the impact are translated as the transferred energy to the sample. In a composite material, this absorbed energy is dissipated by the work necessary to create a fracture, the different phases of the composite, and by the interface created by both materials, as shown in Equation (3) [21]:

(1) $$\;w\;\approx \;w_{i}+w_{f}+w_{m}+\sum w_{fm}$$

where *w* is the total work of fracture, *w_i_* is the work to necessary to produce a fracture in the material, *w_f_* and *w_m_* are the work dissipated by the creation of the fracture by the fiber and the matrix, respectively, and *w_fm_* is the mechanical work of the interactions between fiber and matrix (physical interactions, chemical bonds, etc.). The difference between the results of the notched and un-notched samples allows the calculation of the *w_i_*, which, due to the unique difference in both tests, is the energy required to produce a fracture.

Table 1 shows the experimental results of PA11 and PA11-SGW composites for notched and un-notched samples. PA11 matrix showed a lower Charpy impact strength than other thermoplastics like PP [4,21]. This difference with PP can be principally related to the glass transition temperature (*T_g_*) of PA11. It is well-known that semi-crystalline polymers have higher impact strengths when tests are performed at temperatures above their *T_g_* [22,23]. PA11’s *T_g_* has been determined to be around 50 °C while PP’s is around −10 °C.

It was found that the Charpy impact strength decreased when fiber content increased. It should be noted that the literature shows that the strength of the interface between the polymer matrix and the fibers impacts the behavior of the fracture. In this sense, the quality of the interface and the dispersion of the filler was shown to be significant in other fiber reinforced thermoplastics like PP, where the impact strength values increased when coupling agents were used [4,24,25,26]. Moreover, the presence of SGW fibers contributes to further limiting the mobility of the PA11 chains, making the composites more fragile. Ongoing research shows that this phenomenon occurs also when the temperature is raised, due to the stiffness of the fibers. This effect was also observed in the tensile properties of PA11-SGW composites, where the toughness of the material decreased, showing a similar tendency [12]. However, the obtained results were better than those obtained for coupled cellulose reinforced PP composites (between 18–24 kJ/m^2^) [4,21,27], PP reinforced with 20–30% *w*/*w* glass fiber (GF) (18–23 kJ/m^2^) [26] and some polyamides reinforced with 30% *w*/*w* GF (8–12 kJ/m^2^) [28]. This result can be related to the achievement of a well-balanced fiber-matrix interaction on PA11/SGW system, even avoiding the use of coupling agents.

**Figure 1:**
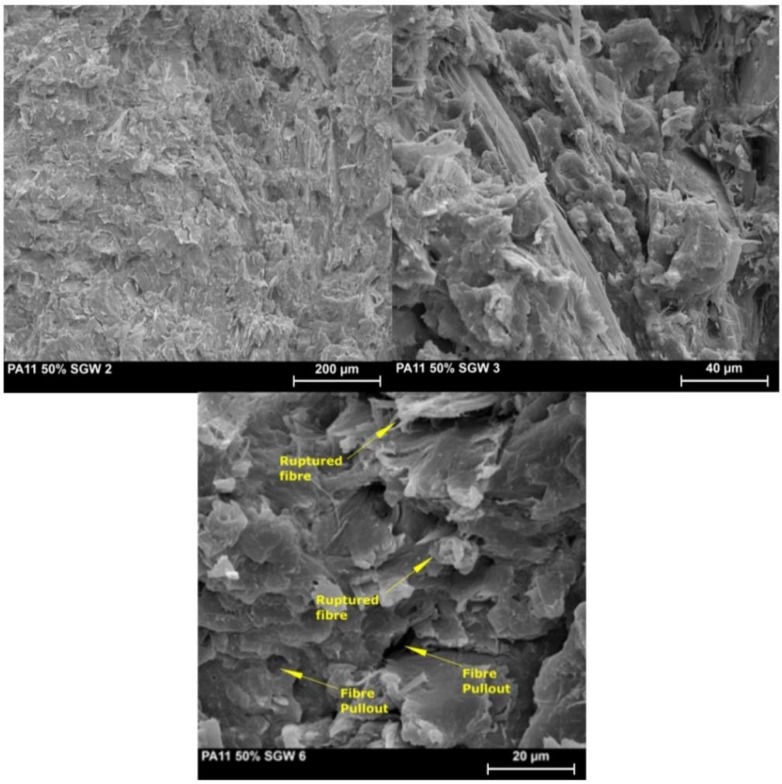
SEM photographs of PA11 + 50%SGW composite at different resolutions.

Figure 1 shows SEM micrographs of PA11 + 50%SGW composite specimens tested under Charpy impact. Some voids, attributed to slip-out fibers during the impact, can be observed. Nevertheless, broken fibers are also found (Figure 1), indicating a not strong but suitable interface between PA11 and SGW fibers [10,12,29].

The un-notched and notched samples manufactured with a 60% content of reinforcement showed works of fracture 68% and 76%, respectively, lower than that of PA11 (Equation (1)). This difference was related with higher energy consumption during fracture initiation (*w_i_*), and also because polyamides are notch sensitive [28,30].

**Figure 2:**
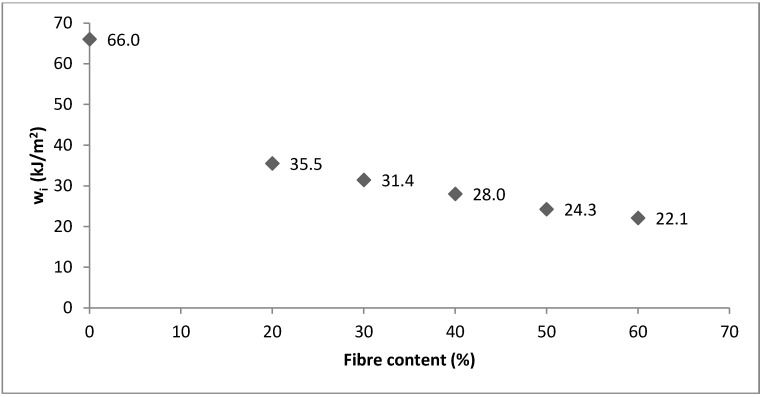
*w_i_* for PA11 and PA11-SGW composites regarding fiber content.

Values of *w_i_* are represented in Figure 2. As shown, the *w_i_* value decreased when fiber content was increased. Nonetheless, it was observed that the tendency was logarithmic instead of linear. This indicated that the presence of SGW in the composite had higher impact than its amount. This was mainly attributed to discontinuities produced by the presence of fibers. Initially, it was expected that higher contents would produce more discontinuities in the polymer matrix. Nonetheless, if the *w_i_* of PA11 is omitted, the results of the composite materials decrease linearly with a small slope (Figure 2). The addition of 20% and 40% *w*/*w* of SGW reduced the work 46% and 58%, respectively, with a difference of 12% between both materials.

### 3.2. Hydrophility and Water Uptake Behavior at 23 °C and 40 °C

It is generally accepted that water acts as plasticizer in polymers. Water penetrates the amorphous phases of the polymer [31], affecting their mobility. Moreover, in some polar polymers like polyamide 6 (PA6), the crystalline phase can be also affected by water absorption [32,33]. Polyamides have a higher polar character than other polymers due to the presence of amide bonds. The capacity of the amide group to establish hydrogen bonds leads PA to absorb more water than other polymer matrixes, since it is necessary to consider not only the adsorption phenomena on the amorphous phase, but also the absorption due to the hydrogen bond capacity.

On the other hand, although lignocellulosic fibers such as SGW have lower hydrophilic behavior than pure cellulose due to lignin presence on the surface of the fiber, their capacity to adsorb/desorb water is huge compared with a PA11 matrix. SGW fibers contain around 27% lignin and only approximately 40% cellulose [34]. Moreover, a high part of the lignin is in the surface; thus, the presence of cellulose in the surface is limited [12]. It is expected that increments of fiber contents in the composite will increase the hydrophilic behavior of the PA11-SGW composites. One simple technique to determine this hydrophilic behavior is to measure the water contact angle. The mean contact angle determined is shown in Table 2.

PA11 achieved an average water contact angle of around 77.1°, indicating its hydrophilic behavior, since hydrophobic materials are considered for angles around 100° [35]. In the case of its composites, the water contact angle decreased to around 68.8° and 65.9° for the composites containing 20 and 50%SGW, respectively. The unexpected higher value found for PA11 + 60%SGW composite (69°) can be related with a lower homogeneity of the samples, making it difficult to correctly characterizae. The PA11 + 60%SGW composite material has previously shown difficulties in mechanical characterization [12]. These difficulties were related to poor wettability of the fibers by the polymer matrix. This poor wettability can produce a low homogeneity of the sample, creating roughness which can impact the contact angle measurement [36].

The values of contact angle are an average of the contact angles observed over 60 s. Although this angle reduced slightly with time, due to the polarity of the samples, it was possible to determine the wetting energy (*E_w_*) using the average angle obtained during the measure. The *E_w_* is defined as the energy required to wet the material, and is considered to be exothermic. *E_w_* was calculated from the contact angle [37] as *E_w_* = *γ cos θ*, where *θ* is the contact angle and *γ* the surface tension of water (72.8 mJ/m^2^). The *E_w_* values obtained for the PA11 and its composites are shown in Table 2. As expected, *E_w_* increased at lower angles.

As mentioned in the methods section, immersion water uptake tests were performed at room temperature (23 °C) and at 40 °C. The tests were carried out under accelerated conditions. Although the *T_g_* of the PA11 and the composites were determined to be around 50 °C, the start of the chains mobility in the storage and loss modulus was around 40 °C [19]. The water uptake profiles obtained from the water uptake test at 23 °C and 40 °C were similar to those obtained for other natural fiber reinforced thermoplastic polymers [3,16,38]. The water uptake capacities increased with the fiber contents. The samples with higher fiber percentages needed less time to reach saturation. In the case of 40 °C, the saturation time was also reduced due to the higher mobility of the amorphous chains of PA11 matrix from the beginning of the test, enhancing the mobility of the water molecules. Moreover, their higher content in the composite implies a higher fiber presence on the surface of the specimen, which can facilitate the dispersion of the water molecules through the material. Nonetheless, the literature reports lower water uptake values for composites with PA11 at similar beech fiber contents [10]. This difference can be related to the aspect ratio; thus, the higher specific surface of SGW fibers with respect to beech fibers, leading to higher availability of cellulose hydroxyl groups, and hence, higher interaction with the water molecules. On the other hand, the water uptake at the saturation point (*M_∞_*) observed for PA11 and PP reinforced with SGW at 50% of reinforcement content was almost the same [3]. The result was unexpected due to the higher hydrophilicity of PA11 and the use of a coupling agent in the PP-SGW formulation. At 20% of SGW content, the result in PA11 is slightly superior and similar to the PP-SGW composite without the use of a coupling agent. Nevertheless, similar results were observed for other PP-cellulose reinforced composites [16].

The water uptake kinetics can be modeled by Fick’s dispersion theory. The experimental results can be linearized by the logarithm of the water uptake (*M_t_*) divided by the *M_∞_*, represented regarding the logarithm of the time (*t*). The regressions of the results yield Equation (2):

(2) $$\;\log\left(\frac{M_{t}}{M_{\infty }}\right)=n\log\left(t\right)+\log K$$

where *n* and *K* are kinetic constants. Another important parameter in the kinetics model is the diffusion coefficient (*D*), obtained from the Fick’s Law and related with the ability of the solvent, in this case, water, to penetrate solid materials. As a higher *D* value indicates higher facility of water to penetrate through the solid. *D* was calculated at low times of immersion, when *M_t_*/*M*_∞_ ≤ 0.5, as:

(3)  MtM∞=4L·(Dπ)12·t12

where *L* are the thickness of the studied samples. The measured values of these constants for 23 and 40 °C are shown in Table 3.

The *n* values for the PA11 matrix and its composites at 23 and 40 °C increased with the fiber contents. However, no considerable differences were obtained between both temperatures. The values of *n*, lower than 0.5, indicated a pseudo-Fickian dispersion case for PA11 and PA11 + 20%SGW composites [39]. Nonetheless, when the fiber contents were increased to 50% and 60%, a shift in the Pseudo-Fickian behavior to a Fickian dispersion case I was observed [16]. A Fickian dispersion case I is related to solvents with lower mobility than the polymers chains, and a pseudo-Fickian behavior is related to a similar mobility of the solvent and the polymer chains. The *n* value is related to the time necessary to reach the saturation point. The slightly differences obtained in the *n* values at both temperatures indicated a high mobility of the PA11 chains, even at the lowest temperatures, probably produced by the high diffusion of the water in the PA11, enhanced by the polar groups. Its decrease when the fiber contents increased can be related to the stiffness of the fibers, inhibiting the polymer chain’s mobility. On the other hand, *K* is a constant, related to the system. The increment of the temperature of the system was reflected as an increment of *K* at 40 °C. The presence of the fibers reduced this constant by around 50% in the composites at 23 °C, and by around 66% at 40 °C, in terms of PA11 constant. A clear dependence of the fiber content on *K* was not obtained, although it can be determined that the reduction of the PA11 chains due to the fibers present had an impact in this constant.

The *D* results obtained for PA11 and its composites showed, in both temperatures, a lower ability of the solvent to penetrate in the 20% and 50% of reinforced composites regarding PA11. This is in concordance with the *n* values obtained and the shift from to pseudo-Fickian to Fickian diffusion. The lower *D* values in the 20% and 50% of SGW reinforced composites were related to the lower mobility of the PA11 chains inhibited by the lignocellulosic reinforcement. Moreover, the hydrogen bond interaction between the fibers and the PA11 can also have some negative effects in the diffusion of the water through the composite. As expected, the *D* values increased with the temperature due to the enhancement in the mobility of PA11 chains with temperature. Nonetheless, the higher effect of the temperature was obtained for the PA11 sample, where the *D* value increased up to 5 times. In the case of the PA11 + 20%SGW and PA11 + 50%SGW, the increase was moderate. Thus, a positive impact was obtained in the composite materials by the fibers, even at temperatures near the *T_g_* of the polymeric phase. The *D* results were in the same range than the obtained for PA11 and other polyamides in the literature [31,40].

Otherwise, the water diffusion through polymer materials is considered to be in the amorphous part of the polymer [31]. Nonetheless, the effect of the water in the crystalline part is not well established [14]. In this sense, the crystalline structure of the PA11 can have some effect on the absorption and diffusion processes. These processes can be also affected by the different structures of PA11, which depend on the hydrogen bond disposition in the space between the PA11 chains. In the studied samples, the predominant phase was the *δ*’ [19]. Nonetheless, other crystalline phases were shown to be more thermodynamically stable [41], which can reduce their *D* coefficient. Nevertheless, more research is required to establish a possible relation.

The *M_∞_* of the materials were slightly higher at 40 °C than at room temperature. These increments were related to the higher temperature of the process. It can be expected that higher *M_∞_* had to be obtained due to the enhancement of the mobility by the plasticizing effect of the water molecules, which decreased the *T_g_* of the materials [40].

In the case of the PA11 + 60%SGW composite, a higher value of *D* than PA11 matrix was obtained at 23 °C. This was related to the previously observed high content of fiber, and the poor wettability of such fiber [12]. A similar effect was observed in PP without the use of a coupling agent, where higher *D* and *M_∞_* were obtained due to the PP not wetting the fiber correctly [3]. At 40 °C, the *D* was lower than PA11, but around twice those obtained with the 20 and 50% reinforced composites. This effect was related to the enhancement in the mobility of the amorphous chains of the PA11, while in the composite material it is more limited due to the stiffness of the SGW fibers. Nevertheless, the presence of voids in the material produced its higher *D* compared to the other composites.

As expected, *D* increased with the temperature. In Fickian dispersion cases, and also in pseudo-Fickian causes, the dependency of *D* regarding the temperature followed an Arrhenius law [16]:

(4) $$\;D=D_{0}e^{\left(\frac{-E_{d}}{RT}\right)}$$

where *D*_0_ is the permeability index, *E_d_* is the starting energy for the diffusion process, *T* is the temperature and *R* is the gas constant. The linearization of the Equation (4) allowed us to calculate the *E_d_* value of PA11 and its composites (Table 4).

The *E_d_* value found for PA11 was in agreement with previous studies with PA11 and other PA [42,43]. A reduction in the *E_d_* was observed in the composite materials regarding the PA11 matrix. This result can be expected, and it is related to the hydrophilic behavior of the fiber, its higher presence in the surface of the specimens, and the reduction of the polymeric matrix content in the composite, which can facilitate the beginning of the diffusion process.

On the other hand, the *E_d_* obtained for PA11 was lower than the activation energy (*E_a_*) calculated for the hydrolysis of PA11 (80–110 kJ/mol) [43,44], indicating no degradation process of the PA11 used in the composites materials at the studied temperatures. This was expected, and the literature reports that the hydrolysis degradation only occurs for temperatures higher than 90 °C. Additionally, the fiber content is not supposed to have any effect in the PA11 hydrolysis, at least at the studied temperatures. Nonetheless, more research at temperatures ranging from 90 to 140 °C, where hydrolysis of the amide group for PA11 is observed, has to be done to verify the effect of the fiber in the hydrolysis of PA11.

## 4. Conclusions

The impact strength and water uptake behavior of PA11 and PA11-SGW were analyzed in this work. The un-notched and notched Charpy test showed a reduction of the PA11 impact strength due to the addition of reinforcements. The crack propagates along the interface between the fibers and the polymer, as it was the weakest phase of these composite materials. This is in concordance with the slip-out fibers observed in the SEM photography’s.

The difference between un-notch and notch samples allowed us to determine the necessary work to produce a fracture in the material. PA11 showed high strength, although the test was carried out on a temperature lower than its *T_g_*. The obtained results were higher than PP-natural fibers, PP-GF and some GF reinforced polyamides composites. Nonetheless, the matrix and the composite samples showed low resistance to the propagation of a fracture. This was related to PA’s notch-sensitive behavior and the discontinuity produced in the matrix by the fibers.

The hydrophilicity of PA11 and PA11-SGW composites was studied by their water contact angle. The results showed a hydrophilic behavior for the PA11 which was enhanced by the addition of SGW fibers. The *E_w_* reflected a more exothermic value for the composites materials in accordance with the observed contact angle.

The water uptake tests were carried out at room temperature (23 °C) and at 40 °C, a temperature near the *T_g_* of PA11. An increase of the *M_∞_* was observed when the fiber content was increased, caused by the hydrophilic behavior of lignocellulosic fibers. Although the hydrophilicity of PA11, the *M_∞_* observed for PA11 and PP composites were similar at high fiber contents. The model of the water uptake curve led to determine some Fick’s parameters. The PA11 matrix showed a pseudo-Fickian diffusion behavior, while the composite materials shifted to a Fickian one. The same behavior was obtained at 23 and 40 °C. *D* showed lower values for PA11 + 20%SGW and PA11 + 50%SGW composites than the obtained for PA11, again for both temperatures. This was related to the reduced mobility of the polymer chains due to the fiber’s stiffness. As expected, the *D* coefficient was increased at 40 °C compared to the 23 °C value. However, any enhancement of the diffusion process was reduced by the presence of fibers. In the case of PA11 + 60%SGW composite, high *D* values were obtained because the fiber was not correctly wetted by the matrix, and some voids were identified, thus enhancing the diffusion. Finally, the *E_d_* for PA11 and PA11-SGW composites were determined. In the composite material, their energy was reduced, favoring the start of the process.

## Figures and Tables

**Table 1:** Charpy impact strength for un-notched and notched PA11-SGW composites samples.

Fiber Content (%)	Charpy Impact Strength	
	Un-Notched (kJ/m^2^)	Notched (kJ/m^2^)
0	77.5 ± 8.5	11.5 ± 1.9
20	40.8 ± 7.5	5.2 ± 0.2
30	35.8 ± 7.7	4.4 ± 0.2
40	31.4 ± 2.9	3.3 ± 0.2
50	27.4 ± 1.4	3.2 ± 0.2
60	24.8 ± 1.2	2.7 ± 0.1

**Table 2:** Average water contact angles and wetting Energy (*E_w_*) for PA11 and PA11 composites.

Sample	Average Contact Angle (°)	*E_w_* (mJ/m^2^)
PA11	77.1 ± 0.4	16.3 ± 0.5
PA11 + 20%SGW	68.8 ± 0.4	26.3 ± 0.5
PA11 + 50%SGW	65.9 ± 0.5	29.7 ± 0.6
PA11 + 60%SGW	69.3 ± 1.3	25.7 ± 1.5

**Table 3:** Fick’s parameters and diffusion coefficient at 23 and 40 °C regarding the fiber content.

Temperature (°C)	Fiber Content (%)	*M_∞_* (%)	*n*	*K*	*D* (10^−13^ m^2^·s^−1^)
23	0	1.43 ± 0.02	0.305 ± 0.045	0.088 ± 0.023	4.95 ± 0.02
	20	3.42 ± 0.03	0.371 ± 0.006	0.04 ± 0.002	2.19 ± 0.01
	50	10.01 ± 0.25	0.428 ± 0.004	0.03 ± 0.001	2.70 ± 0.02
	60	12.51 ± 0.30	0.453 ± 0.002	0.043 ± 0.001	5.81 ± 0.47
40	0	1.58 ± 0.03	0.272 ± 0.007	0.155 ± 0.007	22.00 ± 1.65
	20	4. 55 ± 0.10	0.387 ± 0.010	0.059 ± 0.004	6.02 ± 0.04
	50	11.10 ± 0.20	0.419 ± 0.004	0.057 ± 0.002	8.05 ± 0.06
	60	13.20 ± 0.02	0.481 ± 0.006	0.055 ± 0.003	14.44 ± 0.44

**Table 4:** *E_d_* of PA11 and PA11 composites.

Fiber Content (%)	*E_d_* (kJ/mol)
0	68
20	46
50	49
60	41

## Supplementary Materials

The following are available online at http://www.mdpi.com/2073-4360/10/7/717/s1, Figure S1: Un-notched specimen for Charpy test following ISO 179, Figure S2: Notched specimen for Charpy test following ISO 179.
