# Supplementary material for: Impact Strength and Water Uptake Behaviors of Fully Bio-Based PA11-SGW Composites
Source: Polymers (Basel). 2018 Jun 29;10(7):717. doi: 10.3390/polym10070717 (PMC6404017; doi:10.3390/polym10070717)
Supplement: Supplementary file 1 [file polymers-10-00717-s001.pdf]

Supplementary Materials

Impact Strength and Water Uptake Behaviors of Fully Bio-Based PA11-SGW Composites

Helena Oliver-Ortega [1,*], José Alberto Méndez [1], Francesc Xavier Espinach [2], Quim Tarrés [1], Mònica Ardanuy [3] and Pere Mutjé [1]

1 Group LEPAMAP, Department of Chemical Engineering, University of Girona, C/M. Aurèlia Capmany, 61, 17003 Girona, Spain; jalberto.mendez@udg.edu (J.A.M.); joaquimagusti.tarres@udg.edu (Q.T.); pere.mutje@udg.edu (P.M.)

2 Design, Development and Product Innovation, Department of Organization, Business Management and Product Design, University of Girona, C/M. Aurèlia Capmany, 61, 17003 Girona, Spain; francisco.espinach@udg.edu

3 Department of Materials Science and Metallurgy, Textile Engineering, Polytechnic University of Catalonia, C/Colom, 11, 08222 Terrassa, Spain; monica.ardanuy@upc.edu

* Correspondence: helena.oliver@udg.edu; Tel.: +34-669-996-998

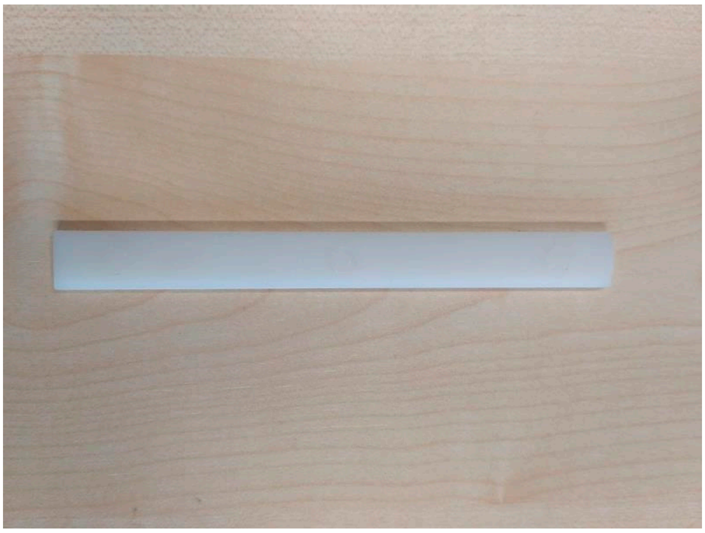

Figure S1. Un-notched specimen for Charpy test following ISO 179.

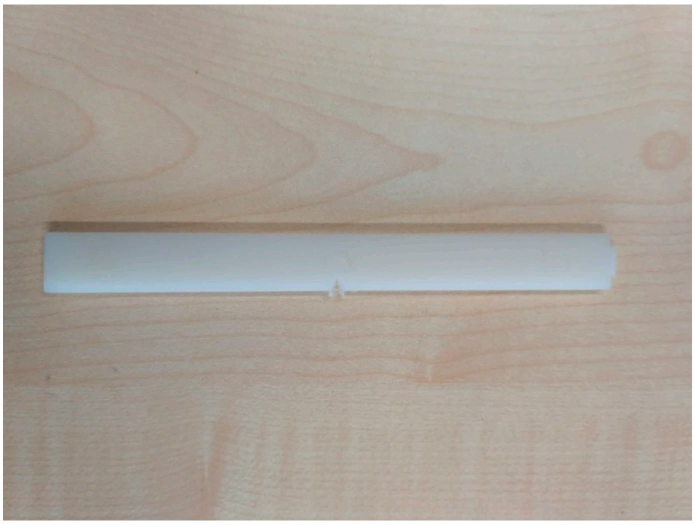

Figure S2. Notched specimen for Charpy test following ISO 179.
